# Contrast-enhanced CT in determining resectability in patients with pancreatic carcinoma: a meta-analysis of the positive predictive values of CT

**DOI:** 10.1007/s00330-016-4708-5

**Published:** 2017-01-16

**Authors:** Inne Somers, Shandra Bipat

**Affiliations:** Department of Radiology, Academic Medical Centre, University of Amsterdam, G1-212, Meibergdreef 9, 1105 AZ Amsterdam, The Netherlands

**Keywords:** Pancreatic Neoplasms, Computed Tomography, Positive Predictive Value, Systematic Review, Surgery

## Abstract

**Objective:**

To obtain a summary positive predictive value (sPPV) of contrast-enhanced CT in determining resectability.

**Methods:**

The MEDLINE and EMBASE databases from JAN2005 to DEC2015 were searched and checked for inclusion criteria. Data on study design, patient characteristics, imaging techniques, image evaluation, reference standard, time interval between CT and reference standard, and data on resectability/unresectablity were extracted by two reviewers. We used a fixed-effects or random-effects approach to obtain sPPV for resectability. Several subgroups were defined: 1) bolus-triggering versus fixed-timing; 2) pancreatic and portal phases versus portal phase alone; 3) all criteria (liver metastases/lymphnode involvement/local advanced/vascular invasion) versus only vascular invasion as criteria for unresectability.

**Results:**

Twenty-nine articles were included (2171 patients). Most studies were performed in multicentre settings, initiated by the department of radiology and retrospectively performed. The I^2^-value was 68%, indicating heterogeneity of data. The sPPV was 81% (95%CI: 75-86%). False positives were mostly liver, peritoneal, or lymphnode metastases. Bolus-triggering had a slightly higher sPPV compared to fixed-timing, 87% (95%CI: 81-91%) versus 78% (95%CI: 66-86%) (*p* = 0.077). No differences were observed in other subgroups.

**Conclusions:**

This meta-analysis showed a sPPV of 81% for predicting resectability by CT, meaning that 19% of patients falsely undergo surgical exploration.

***Key points*:**

• *Predicting resectability of pancreatic cancer by CT is 81*% (*95*%*CI*: *75*-*86*%).

• *The percentage of patients falsely undergoing surgical exploration is 19*%.

• *The false positives are liver metastases*, *peritoneal metastases*, *or lymph node metastases*

**Electronic supplementary material:**

The online version of this article (doi:10.1007/s00330-016-4708-5) contains supplementary material, which is available to authorized users.

## Introduction

Contrast-enhanced computed tomography (CT) plays a central role in staging of pancreatic cancer [1]. The staging system depends on the tumour size and location, extension beyond the confinement of the pancreas, adjacent vessels contact or encasement/occlusion, and the presence of distant metastatic disease (e.g. lungs, liver, bones, lymph nodes, and peritoneum). The presence of distant metastatic disease excludes patients from curative resection intent. In addition, based on which vessel is involved and the degree of involvement, patients are excluded or included from curative resection intent. In two recently published reviews [1, 2], one comparing CT with EUS showed lower diagnostic values for CT in evaluating vascular invasion (sensitivity 63% and 72% and specificity 92% and 89%) and the other review summarizing CT findings showed high sensitivity and specificity of 77% and 81%, respectively. Although the sensitivity value in the review by Yang et al is low, CT remains the most commonly used modality, because of the evaluation of distant metastases in a one-stop-shop strategy [3–6].

However, there are concerns that in patients with (borderline) resectable tumours on CT, a substantial number of patients (40%) are found to be unresectable during surgical exploration, based on the presence of either vascular invasion or metastatic disease [7]. This means that unnecessary surgical exploration/laparotomy is performed in 40% of these patients. In a recent review the additional role of laparoscopy before surgical exploration can reduce this percentage to 20% [7]. Several studies also showed the significant role of additional PET-CT to reduce the number of unnecessary surgical explorations [8–11]. However, both PET-CT and laparoscopy are operationally high cost and invasive methods. Therefore, CT remains playing a major initial role in the overall staging of pancreatic cancer

The most important outcome in determining resectability by CT is the positive predictive value for resectability, as in general the patients with (borderline) resectable tumours on CT will undergo surgical exploration followed by resection.

In patients found to be unresectable on CT it is not ethical to perform an invasive technique, and in addition abovementioned reviews [1, 2] showed that CT has a low number of patients with false positive vascular invasion. As this is an important feature in determining unresectablity, the number of patients falsely judged to be unresectable by CT is very low with high predictive values for unresectablity between 89-100% [3]. Therefore, summarizing the predictive values for unresectablity seems not to be relevant.

The aim of this study is to obtain a summary positive predictive value of contrast-enhanced CT in determining the resectability and to report the incidence of contra-indicative factors in the false positive patients (patients judged to be resectable on CT while on reference standard these patients were found to be unresectable).

## Materials and methods

### Search strategy

Computerized searches in MEDLINE and EMBASE databases from JAN 2005 to JUN 2015 were performed to identify relevant abstracts. For MEDLINE the following keywords were used: "Pancreatic Neoplasms"[Mesh] ”AND ("Tomography, X-Ray Computed"[Mesh] OR "Multidetector Computed Tomography"[Mesh] OR "Four-Dimensional Computed Tomography"[Mesh] OR "Spiral Cone-Beam Computed Tomography"[Mesh] OR "Cone-Beam Computed Tomography"[Mesh] OR "Tomography Scanners, X-Ray Computed"[Mesh] OR "Tomography, Emission-Computed"[Mesh] OR "Tomography, Spiral Computed"[Mesh] OR "Tomography, Emission-Computed, Single-Photon"[Mesh]).

For EMBASE the following text words were used: pancreatic cancer (tw) AND computed tomography (tw) or *computer assisted tomography (tw).

### Selection of eligible articles

All retrieved hits were evaluated by a reviewer (X1), with experience in data-extraction of 20 meta-analyses on diagnostic accuracy studies. All titles and abstracts were screened in four steps.

Step 1: duplicate papers, letters/comments/editorial/conference abstracts, not pancreatic cancer relevant papers and papers describing other type of pancreatic cancer were excluded.
Step 2: case reports, studies on animals/cell lines/phantoms/children, reviews/guidelines, studies only evaluating treatment, studies evaluating prognosis/survival (not imaging related), studies clearly evaluating other imaging (biopsy, FNA), and studies reported in Chinese, Korean, Japanese, and Russian were subsequently excluded.
Step 3: subsequently studies were excluded if other parameters based on CT were evaluated; CT was only used for screening or response monitoring or recurrent/follow-up or prognosis/association.
Step 4: To identify additional studies, reference lists of relevant articles were checked manually and additional search was performed between JUN 2015 and DEC 2015.

### Inclusion and exclusion criteria

All potentially eligible articles were double-checked on inclusion and exclusion criteria by the same reviewer with a delay of 4 weeks. Inclusion criteria were: (a) more than 25 patients with pancreatic adenocarcinoma, (b) patients undergoing contrast-enhanced CT, (c) contrast-enhanced CT for determining resectability, (d) histopathology (surgery, biopsy, or cytology), laparoscopy/laparoscopic ultrasound, follow-up, or consensus were used as reference test, (e) absolute numbers of true positive and false positive results available or could be extracted. Exclusion criteria were: (a) data on same outcome of same study population (study with the largest population was included) and (b) results on combination of different imaging modalities presented and cannot be differentiated for contrast-enhanced CT.

### Data extraction

Of the included papers, data were extracted by two reviewers independently (X1 and X2, radiologist with experience in MRI imaging of the pancreas) and discrepancies were resolved by consensus. The following data (including quality assessment) were extracted:

#### Study design

Year of publication, study period, country of origin, setting (single-centre/multicentre), department of first author, type of data-collection (prospective/retrospective/unclear), and whether ethical approval was obtained (yes/no/unclear).

#### Patient characteristics

Patient population (suspected/diagnosed/underwent surgery), inclusion/exclusion criteria, number of patients with pancreatic carcinoma included/analysed, number of patients included in total in study, distribution of total number of patients (carcinoma, other malignant lesions, benign lesions), age of patients (mean + SD or median + range), sex ratio (male: female), number of patients undergoing surgery/resection.

Quality criterion was whether consecutive/random sample of patients were enrolled (yes/no/unclear) [12].

#### Imaging techniques

Bowel preparation, intravenous contrast agent (type, concentration, and amount), phased used (scan delay and reconstructed slice width if available).

Quality criterion was whether the execution of CT was described in sufficient detail to permits its replication (yes/no/unclear) [12]. The execution of CT was described in sufficient detail if type of scanner and type, amount and concentration of contrast agent and phases with scan delay were described

#### Image evaluation

Method of reconstructions (e.g. multiplanar reconstruction), observers (number, experience, and data on interobserver analysis), and CT criteria used for resectability/unresectablity. Quality criteria were: 1) whether the interpretation of CT was described in sufficient detail to permit its replication (yes/no/unclear). The interpretation of CT was described in sufficient detail if method of reconstruction, number/experience of observers, and criteria for resectability/unresectablity were given; 2) whether CT results were interpreted without knowledge of the results of the reference standard (yes/no/unclear); and 3) if resectability/unresectability criteria were pre-specified (yes/no/unclear) [12].

#### Reference standard

Data on composition of the reference standard (surgery, resection/histopathology, biopsy/aspiration, laparoscopy with or without ultrasound, follow- up, consensus) were extracted. Quality criterion was whether the reference standard was likely to correctly classify the target condition (yes/no/unclear). In case of consensus including CT, the reference standard was assessed as not correctly classifying the target condition.

#### Time interval between CT and reference standard

The time interval between CT and reference standard was recorded. Quality criterion was whether there was an appropriate interval [<1 month for surgery, resection/histopathology, biopsy/aspiration, laparoscopy with or without ultrasound, and < 12 months for follow-up between CT and reference standard (yes/no/unclear)] [12].

#### Data on resectability/unresectability

We extracted data by using CT resectability categories as were reported in the articles versus the two reference standard categories. The data was, therefore, expressed as follows:

- 2 × 2 table (resectable OR unresectable on CT vs. resectable OR unresectable on references standard).
- 1 × 2 table (resectable on CT vs. resectable OR unresectable on references standard).

True positive is defined as resectable on both CT and reference standard.

False positives are defined as resectable on CT, while unresectable on reference standard.

Of all false positive resectable tumours (judged to be resectable on CT while unresectable on reference standard), the contraindicative factors were recorded if available.

### Data-analysis

#### Publication bias

To study publication bias, we constructed funnel plot for the positive predictive value (PPV) and the Egger regression test was used to examine funnel plot asymmetry [13]. PPV was defined as TP (resectable on both CT and reference standard)/TP + FP (total number of resectable patients on CT). We placed the PPV on the x-axis and the sample size on the y-axis. A *p*-value of < 0.05 was considered as showing significant publication bias.

#### Summary positive predictive value

The *I* ^2^ statistic, including 95% confidence intervals (CI), was used for quantification of heterogeneity of the PPV [14, 15]. We used either nonlinear fixed-effects (*I* ^2^ ≤ 25%) or random-effects (*I* ^2^ > 25%) approach to obtain summary PPV. Mean logit PPV with corresponding standard errors were obtained, and then antilogit transformation was performed to calculate summary estimates of PPV (sPPV) including 95% confidence intervals (95%CI) [16, 17].

#### Exploratory analysis

To study effect of several quality items on the summary PPV (sPPV), we incorporated these items in the model: (1) publication year; (2) design (multicentre vs. single-centre); (3) department of first authors (radiology vs. other); (4) data collection (prospective vs. retrospective/unclear); (5) patient selection (consecutive vs. not consecutive/unclear); (6) detailed description of CT techniques (yes vs. no/unclear); (7) detailed description of CT interpretation (yes vs. no/unclear); (8) blinded interpretation of CT (yes vs. no/unclear); (9) CT criteria described (yes vs. no/unclear); (10) appropriate interval between CT and reference standard (yes vs. no/unclear); and (11) reference standard correctly classify target condition (yes vs. no/unclear). Year of publication was explored as continuous factor, and all other factors were explored as binomial. We considered factors to be explanatory if the corresponding regression coefficients were significantly different from zero, meaning that *p*-values were less than 0.05.

#### Subgroup analysis

The following subgroups were defined a priori:

1. Studies using bolus triggering versus studies using fixed timing.
2. Studies including both pancreatic and portal phases versus studies including only portal phase.
3. Studies taking all criteria (liver metastases, other distant metastases, lymph node involvement, local advanced and vascular invasion) into account as criteria for unresectablity versus studies taking only vascular invasion as criteria for unresectablity.

The *z* test was performed to analyse differences in logit PPV estimates between subgroups. All data analyses were performed by using software (Microsoft Excel 2000, Microsoft, Redmond, Wash; SPSS 10.0 for Windows, SSPS, Chicago, IL, USA; SAS 9.3, SAS Institute, Cary, NC, USA).

## Results

### Search strategy and selection of eligible articles

**Fig. 1:**
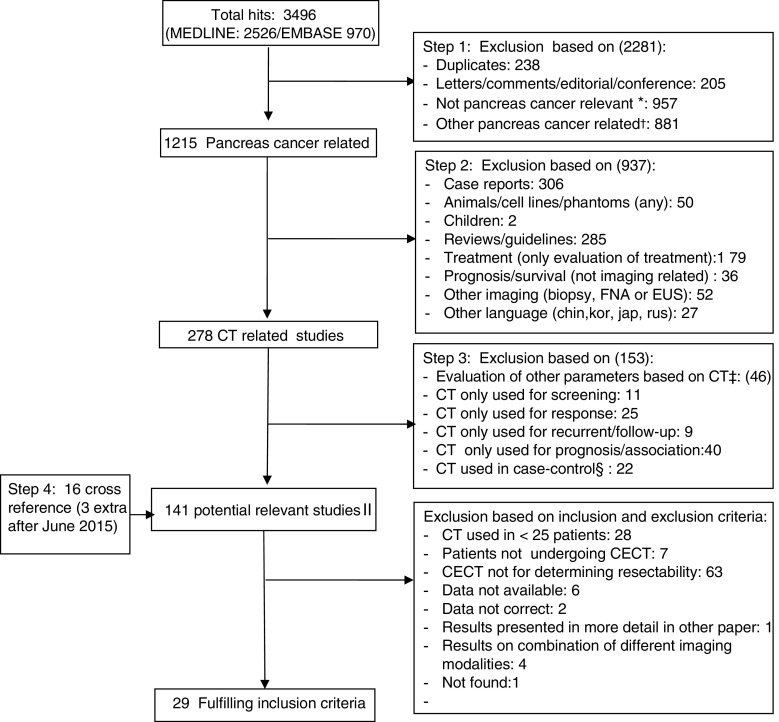
Search, selection and inclusion of relevant papers. *Not relevant (other disease, pancreatitis, lung cancer, ovarian cancer, lipoma, neuroendocrine, insulinoma, melanoma, colon carcinoma, renal cell carcinoma, myeloma, colorectal cancer, pancreatitis, endocrine, intracranial, colitis, hernia, polyposis.). †Other type of pancreatic cancer: pseudopapillary, cystadenoma, acinar. ‡Evaluation of other parameters using CT (e.g. qualitative analysis, tumour volume, interobserver). §Case-control studies: evaluation of techniques in patients with pancreatic cancer vs. control (healthy or pancreatitis or other type of tumour, predefined). II Potentially relevant studies: evaluation of CT in patients with suspected, diagnosed pancreatic cancer

The search strategy resulted in 3496 articles. After excluding all non-relevant papers, 125 articles were found to be potentially relevant. An additional 16 articles were found after manually cross-checking, resulting in 141 relevant articles. These articles were checked on inclusion and exclusion criteria. Finally, 29 [18–46] fulfilled the inclusion criteria and data-extraction was performed. The search strategy and selection of eligible articles are shown in Supplement 1 and Fig. 1. The excluded articles are presented in Supplement 1.

### Study design

Most studies were performed in multicentre setting, initiated by the department of radiology and retrospectively performed. In case a study was prospectively designed [22, 30, 37, 41], resectability by CT was mostly retrospectively analysed. All data on study design characteristics are shown in Table 1.

**Table 1:** Study design characteristics of the included studies

Study author	Year of publication/Journal	Study period	Country of origin*	Type of study†	Department first author	Type of data collection	Ethical approval
Ellsmere [18]	2005/Surg Endosc	Jan 1999-March 2002	USA	Multi-centre	Surgery	Retrospective	UNCLEAR
Imbriaco [19]	2005/AJR Am J Roentgenol	Sept 2001-Feb 2003	Italy	Multi-centre	Radiology	Prospective	YES (informed consent)
Karmazanovsky [20]	2005/Abdom Imaging	1994-2003	Russia	Single-centre	Radiology	Retrospective	UNCLEAR
Li [21]	2005/J Comput Assist Tomogr	Dec 2001-Feb 2004	China	Single-centre	Radiology	Retrospective	UNCLEAR
Phoa [22]	2005/J Surg Oncol	Feb 1997-Jul 1999	The Netherlands	Single-centre	Radiology	Prospective study (retrospective evaluation)	UNCLEAR
Imbriaco [23]	2006/Radiol Med	Sept 2001-March 2004	Italy	Single-centre	Radiology	Unclear	UNCLEAR
Tamm [24]	2006/Abdom Imaging	Not available	USA	Single-centre	Radiology	Retrospective	YES
Kala [25]	2007/Eur J Radiol	Not available	Czech Republic	Multi-centre	Surgery	Retrospective	UNCLEAR
Olivie [26]	2007/JOP	Feb 2003-Jun 2004	Canada	Multi-centre	Radiology	Prospective	YES (informed consent)
Smith [27]	2007/Pancreas	March 2002-March 2005	UK	Single-centre	Radiology	Retrospective	UNCLEAR
Zamboni [28]	2007/Radiology	March 2003-March 2006	USA	Single-centre	Radiology	Retrospective	YES
Furukawa [29]	2008/Arch Surg	Sept 2002-March 2005	Japan	Single-centre	Radiology	Prospective	YES
Klauss [30]	2008/Pancreatology	March 2005-Aug 2006	Germany	Multi-centre	Radiology	Prospective (retrospective evaluation)	YES (informed consent)
Shah [31]	2008/J Surg Res	NA	USA	Single-centre	Surgery	Retrospective	YES
Manak [32]	2009/Abdom Imaging	Jan 2000-Jul 2005	Germany	Multicentre	Radiology	Retrospective	UNCLEAR
Park [33]	2009/J Magn Reson Imaging	Jan 2004-Jul 2008	Korea	Multi-centre	Radiology	Retrospective	YES
Satoi [34]	2009/Hepatogastroenterology	Jan 2000-Apr 2005	Japan	Single-centre	Surgery	Retrospective	YES (informed consent)
Croome [35]	2010/Can J Surg	Jan 2005-Dec 2006	Canada	Multi-centre	Surgery	Retrospective	YES
Grieser [36]	2010/Acta Radiologica	Jan 2002- Jan 2007	Germany	Single-centre	Radiology	Retrospective	YES
Grossjohann [37]	2010/Scand J Gastroenterology	Dec 2005-Dec 2007	Denmark	Multi-centre	Radiology	Prospective (retrospective evaluation)	YES
Kaneko [38]	2010/J Comput Assist Tomogr	Jan 2000-March 2009	USA	Multi-centre	Radiology	Retrospective	YES
Lee [39]	2010/Eur J Radiol	Jan 2003- Jun 2005	Korea	Multi-centre	Radiology	Retrospective	UNCLEAR
Koelblinger [40]	2011/Radiology	Sept 2006-Nov 2007	Austria	Multi-centre	Radiology	Prospective (IC)	YES
Fang [41]	2012/Pancreatology	Nov 2008-Aug 2010	China	Multi-centre	Surgery	Prospective (retrospective evaluation)	YES
Khattab [42]	2012/The Egyptian Journal of Radiology and Nuclear Medicine	Dec 2009-Aug 2011	Egypt	Single-centre	Radiology	Prospective (IC)	YES
Yao [43]	2012/ANZ J Surg	Dec 2006-Jul 2009	Australia	Multi-centre	Surgery	Retrospective	UNCLEAR
Cieslak [44]	2014/Pancreatology	Jan 2007-Dec 2010	The Netherlands	Multi-centre	Gastroenterology	Retrospective	UNCLEAR
Hassanen [45]	2014/The Egyptian Journal of Radiology and Nuclear Medicine	Oct 2010-March 2013	Egypt	Single-centre	Radiology	Prospective (IC)	YES
Iscanli [46]	2014/Turk J Gastroenterol	NA	Turkey	Single-centre	Radiology	Retrospective	YES

^*^Country of origin of first author. † Implies involvement of authors form different centers, not particular the involvement of patients form different centers

### Patient characteristics

In most studies, patients with known pancreatic adenocarcinoma were included. In total, 2171 patients were included (fulfilling inclusion and exclusion criteria) or formed the study group, with ages ranging from 15 to 84 years with a pooled mean of 63.5 years. In 25 studies the distribution of male:female ratio was given as 1088:800.

In some studies [19, 28, 30, 36, 40, 41, 43, 44] data on other malignancies were also given; however, as most of the patients had adenocarcinoma, we did not exclude these studies. All patient characteristics per study are given in Table 2.

**Table 2:** Patient characteristics of the included studies

Study author	Patient Population (suspected or known)	Selection criteria (inclusion and/or exclusion criteria)	Number of total patients included*	Distribution of patients (adenocarcinoma; other malignancies; benign lesion)	Age (mean or median and SD or range) in years	Sex ratio (male: female)	Consecutive or random selection
Ellsmere [18]	Known	Inclusion: Patients with a diagnosis of pancreatic head adenocarcinoma who underwent a gastrointestinal procedure. Exclusion: Patients who did not undergo a thin-section dual-phase MDCT and attempted pancreaticoduodenectomy at the institution	44	Adenocarcinoma: 44	Not available	Not available	YES
Imbriaco [19]	Suspected	Inclusion: Patients with a suspected pancreatic mass based on clinical symptoms, laboratory findings, and results of ERCP or sonography	71	Adenocarcinoma: 37 Other malignancy: 3 Benign lesions: 31	Mean: 63 ± 12 Range:29-80	41:30	YES
Karmazanovsky [20]	Known	Inclusion: Patients who had morphologically confirmed pancreatic head adenocarcinoma by using spiral CT with bolus intravenous contrast enhancement; Only patients who underwent intraoperative surgical exploration were included in this investigation.	89	Adenocarcinoma:89	Mean: 60 Range: 23-80	52:37	UNCLEAR
Li [21]	Suspected	Inclusion: Patients with presumed pancreatic cancer underwent surgery	54	Adenocarcinoma: 54	Mean: 61.2 Range: 40-79	37:17	YES
Phoa [22]	Suspected	Inclusion: Patients suspected of having pancreatic carcinoma who underwent a spiral CT for staging; Patients with pancreatic head carcinoma eventually who underwent surgery for attempted resection with curative intent. Exclusion: patients with cholangiocarcinomas, cystic tumours or endocrine tumours.	71	Adenocarcinoma: 71	Median: 62 Range: 42-76	33:38	YES
Imbriaco [23]	Suspected	Patients with suspected pancreatic cancer based on clinical and laboratory findings and on results of endoscopic retrograde cholangiopancreatogram (ERCP) or ultrasonography (US).	78	Adenocarcinoma:46 Benign lesions: 32	Mean ± SD: 64 ± 12	42:36	UNCLEAR
Tamm [24]	Known	Inclusion: Patient with biopsy-proven adenocarcinoma of the pancreas who underwent MDCT imaging using a dual-phase pancreas protocol and endoscopic ultrasound. Exclusion: patients with other histology or who underwent CT scanning using other imaging regimens were not included. Also excluded were patients who were identified as having resectable disease but refused surgery, were lost to follow-up or had significant comorbid disease that precluded surgery.	55	Adenocarcinoma: 55	Mean: 67 Range: 46–86	31:24	UNCLEAR
Kala [25]	Known	Inclusion: Patients with pancreatic cancer.	55 (undergoing CT)	Adenocarcinoma: 55	Not available	Not available	UNCLEAR
Olivie [26]	Suspected or known	Inclusion: Patients referred with a known or suspected diagnosis of cancer of the head and patients found to be resectable.	28 (study group)	Adenocarcinoma:28 (study group)	Mean: 63 Range: 40-76	14:14	YES
Smith [27]	Known	Inclusion: Patients with pancreatic ductal adenocarcinoma. Exclusion: Patients with ampullary tumours, tumours of the body and tail of the pancreas, and benign disease	33 (underwent surgery)	Adenocarcinoma: 33 (study group)	Mean: 67.2 Range: 28- 88 (33 patients)	20:13 (33 patients)	YES
Zamboni [28]	Known	Inclusion: Patients who underwent surgical staging or attempt at curative resection for pancreatic carcinoma at our institution; Patients who underwent scanning in our institution with multiphase multidetector CT with multiplanar reconstructions and two- and three-dimensional CT angiography. Exclusion: all patients who underwent scanning at other institutions or with different CT protocols.	114	Adenocarcinoma: 110 Other malignancies: 4	Mean:65.9 Range: 33-85	52: 62	UNCLEAR
Furukawa [29]	Known	Inclusion: Patients were referred for treatment of invasive ductal carcinoma of the pancreas. Exclusion: patients with other histologic types of pancreatic tumour such as endocrine tumour, intraductal papillary mucinous tumour, and solid and pseudopapillary tumour. Patients who already received surgical or medical treatment at another hospital.	213	Adenocarcinoma: 213	Mean: 64 Range: 32-82	136: 77	YES
Klauss [30]	Suspected	Inclusion: Patients with strong clinical suspicion of pancreatic carcinoma based on icterus, a preceding CT or ultrasound scan, unspecific weight loss, or an elevated CA19-9. Exclusion: Patients with renal impairment with creatinine values 12 mg/dL, manifest hyperthyroidism, known contrast medium allergy, and failure to consent to the study.	80	Adenocarcinoma: 36 Other malignancy: 9 Benign lesions: 35	Mean: 64.9 Range: 37–89	43:37	UNCLEAR
Shah [31]	Known	Inclusion: Patients with pancreatic adenocarcinoma Exclusion: Patients presented with other pancreatic neoplasms, such as ampullary adenocarcinoma, pancreatic endocrine tumours, or cystic neoplasms.	88	Adenocarcinoma: 88	Not available	Not available	YES
Manak [32]	Known	Inclusion: Patients with pathologically proven pancreatic adenocarcinoma underwent MDCT and who underwent surgery. Excluded: Patients who received a course of radio-chemotherapy before surgery.	48	Adenocarcinoma: 48	Mean: 64.7 Range: 43–89	26:22	UNCLEAR
Park [33]	Known	Inclusion: Patients with pancreatic cancer who had undergone curative or palliative surgery; Patients with both preoperative contrast-enhanced MRI including MRCP and triple phase MDCT within a month before surgery; Patients with diagnosed pancreatic ductal carcinoma on pathology examination of a surgical specimen.	54	Adenocarcinoma: 54	Mean: 63.1 Range: 28–83	32:22	UNCLEAR
Satoi [34]	Known	Inclusion: Patients with ductal adenocarcinoma after clinical; diagnosis of pancreatic cancer using ultrasonography, CT, MRCP, ERCP, endoscopic ultrasonography, cytological examination of the bile juice and/or biopsy of the bile duct mucosa. Exclusion: endocrine tumour of the pancreas, intraductal papillary mucinous cancer, acinar cell carcinoma, or anaplastic cancer.	80 (patients undergoing multislice CT)	Adenocarcinoma: 80	Mean: 65 Range 39-83	37:43	YES
Croome [35]	Suspected	Inclusion: Patients referred to pancreatic surgeons because of suspected cancer of the pancreatic head.	96 (undergoing CT)	Not available	Not available	Not available	UNCLEAR
Grieser [36]	Known	Inclusion: Patients who underwent surgical exploration or resection of a pancreatic mass at our medical center; patients who underwent a preoperative triphasic MDCT examination (4–64-slice MDCT) performed at our clinic; a detailed report of the operation and a histopathological analysis available.	105	Adenocarcinoma: 60 Other malignancies: 10 Benign lesions: 35	Mean ± SD: 58 ± 15	73:32	YES
Grossjohann [37]	Known/suspected	Inclusion: patients with pancreatic head tumours and with suspected pancreatic head tumours.	49	Adenocarcinoma: 44 Benign lesions: 5	Mean: 66 years Range: 42–83	26:23	YES
Kaneko [38]	Known	Inclusion: Patients presenting to our institution with adenocarcinoma of the pancreatic head were included. Exclusion: located in the pancreatic body or tail were excluded, as well as all patients with other neoplastic/inflammatory processes of the pancreas.	109 (underwent surgery)	Adenocarcinoma: 109	Mean: 65.1 Range: 39-86	56:53	YES
Lee [39]	Known	Inclusion: Patients with newly diagnosed pancreatic ductal adenocarcinoma and who underwent surgery.	56	Adenocarcinoma: 56	Mean: 60.9 Range: 37-76	30: 26	UNCLEAR
Koelblinger [40]	Suspected	Inclusion: Patients suspected of having pancreatic cancer referred to hepatobiliary-pancreatic surgeons; suspected of having pancreatic cancer on the basis of findings from clinical examination (e.g., jaundice, increased CA 19-9 levels, rapid weight loss or previous US or CT studies performed).	89	Adenocarcinoma: 43 Other malignancies: 8 Benign lesions: 38	Mean ± SD: 65.5 ± 10.7	41:48	YES
Fang [41]	Diagnosed	Inclusion: Patients with confirmed pancreatic and periampullary neoplasms.	80	Adenocarcinoma: 57 Other malignancy: 23	Mean ± SD: 57.9 ± 1.7 Range: 15-91	49:31	UNCLEAR
Khattab [42]	Suspected	Inclusion: Patients with clinical and sonographic findings that raised suspicions of pancreatic cancer were included in our study.	39	Adenocarcinoma: 39	Mean: 58.3 Range: 44-73	29:10	UNCLEAR
Yao [43]	Known	Inclusion: Patients with potentially resectable pancreatic tumours detected on contrasted CT imaging and who also had preoperative PET/CT scans.	36	Adenocarcinoma: 30 Other malignancies: 3 Benign lesions: 4 1 patient had both adenocarcinoma and other malignancy	Median: 71 Range: 32–84	24:12	UNCLEAR
Cieslak [44]	Suspected	Inclusion: Patient who underwent exploratory laparotomy for a suspected pancreatic or periampullary malignancy; Patients who underwent both preoperative contrast enhanced CT and endoscopic ultrasonography.	86	Adenocarcinoma: 37† Other malignancy: 30† Benign lesions: 9†	Mean ± SD: 65 ± 10.5	49:37	YES
Hassanen [45]	Suspected	Inclusion: patients with suspected pancreatic carcinoma underwent biphasic MDCT for pancreatic examination.	47 (study group)	Adenocarcinoma: 47	Mean: 63.5 Range: 45-82	32:15	YES
Iscanli [46]	Known	Inclusion: Patients with pancreatic adenocarcinoma confirmed by surgery-pathology or clinical follow-up.	124	Adenocarcinoma: 124	Mean: 60.2 Range: 28-84	83: 41	YES

^*^Fulfilling inclusion criteria or forming the study group.

^†^Number of patients who were found to be resectable

### CT technical features

The number of detectors ranged from 1 to 64. In several studies the specification of CT [18, 35, 43] was not reported. In most of studies, no information on oral contrast administration was given. Intravenous contrast was clearly given in most studies ranging from 90-200 mL. The timing of scanning was either fixed (mostly old studies) or bolus triggering. Pancreatic (late arterial, early portal) and portal (late portal) phases were performed in almost all studies, except in four studies [19, 22, 23, 44]. In ten studies execution of the CT was not described in sufficient detail due to missing information on type of scanner, the type/amount/concentration of iv contrast, and the different phases with scan delay [18, 20, 22, 25, 31, 34, 35, 37, 43, 44]. All details on CT technical features per study are given in Table 3.

**Table 3:** CT technical features of the included studies

Study author	Type of scanner	Bowel preparation	Intravenous contrast agent	Phases	Execution described in detail*
Ellsmere [18]	Not available	Not available	100–150-mL Ultravist	Pancreatic phase: 40-s post-contrast delay, reconstruction slice thickness 3 mm. Portal venous phase: 70-s post-contrast delay, reconstruction slice thickness 5 mm.	NO
Imbriaco [19]	4-slice	10-15 min before CT exam: 500 mL water orally	150 mL Ultravist 370 (Iopromide)	Portal venous phase: 60-s post-contrast delay, 1.25-mm reconstruction interval	YES
Karmazanovsky [20]	Single-slice	Not available	100 mL	***Bolus contrast enhancement*** Arterial phase: 25-s delay Portal vein phase: 80-s delay	NO
Li [21]	4-slice	Not available	120 mL Ultravist 350 (Iopromide)	Arterial phase: 20-s post-contrast delay, 2.5 mm collimation Pancreatic phase: 45-s post-contrast delay, 2.5 mm collimation Hepatic phase: 80-s post-contrast delay, 2.5 mm collimation	YES
Phoa [22]	2-slice	Not available	130 mL Omnipaque 300	Pancreatic phase: 50-s post-contrast delay, collimation 2.5 mm	NO
Imbriaco [23]	4-slice	500 mL water orally	150 mL (370 mg I/mL)	Portal venous phase: 60-s post-contrast delay, reconstruction interval 1.25 mm	YES
Tamm [24]	4-slice	Not available	150 mL Optiray 300 (Ioversol)	Pancreatic phase: 25-s post-contrast delay, 2.5 mm slice thickness and reconstruction to 1.25 mm contiguous images. Portal phase: 55-s post-contrast delay, 5 mm slice thickness and reconstruction to 2.5 mm contiguous images.	YES
Kala [25]	single-slice	Not available	125 mL Optiray 350	Not available	NO
Olivie [26]	16-slice	500 mL of water orally	150 mL Omnipaque 350	Arterial phase: 20-s post-contrast delay, section on width of 2.5 mm and an interval reconstruction of 1.25 mm. Late arterial phase: 40-s post-contrast delay, section on width of 2.5 mm and an interval reconstruction of 1.25 mm Portal phase: 60-s post-contrast delay, section on width of 2.5 mm and an interval reconstruction of 1.25 mm	YES
Smith [27]	4- or 8-slice	Not available	100 mL Omnipaque 300	Pancreatic phase: 40-s post-contrast delay, collimation 2.5 mm; reconstruction overlap, 1.25 mm Portal-venous phase: 65-s post-contrast delay, collimation 2.5 mm, reconstruction overlap 1.25 mm	YES
Zamboni [28]	4-, 8-, 16-, 64- slice	Not available	150–200 mL Optiray 350 (Ioversol)	***Bolus tracking*** **:** 150-HU threshold of enhancement abdominal aorta at the origin of the celiac axis Late arterial–early portal venous phase: 15- s post-threshold delay, collimation 1.25 mm for 4- and 8-slice CT, 0.625 mm for 16-slice or 0.5 mm for 64-slice CT. Venous phase: 25-s after arterial phase, collimation 1.25 mm for 4- and 8-slice CT, 0.625 mm for 16-slice, or 0.5 mm for 64-slice CT.	YES
Furukawa [29]	16-slice	Not available	150 mL Iopamiron (Iopamidol)	Early arterial phase: 20-s post-contrast delay Late arterial phase: 40-s post-contrast delay Pancreatic phase: 70-s post-contrast delay Delayed phase: 120-s post-contrast delay 1-mm section thickness and reconstructed at 1-mm intervals (0.5-mm overlap)	YES
Klauss [30]	16-slice	1.5 L still water	120 mL Ultravist 370	***Bolus threshold*** **:** 100-HU enhancement in the aorta at the level of the superior mesentery artery Arterial phase: 8-s post-threshold delay, reconstructed slice thickness 2/1 Portal venous phase: 35-s post-threshold delay, reconstructed slice thickness (3/3 first and 1/0.5 s)	YES
Shah [31]	64-slice	Water	Intravenous contrast (type/concentration not available	Arterial phase Pancreatic phase Hepatic venous phase	NO
Manak [32]	4- and 16-slice	800 mL water orally with 40 mg butylscopolamin or 1 mg glucagon	120 mL (300–370 mg iodine/mL)	Pancreatic phase: 35-s post-contrast delay, section thickness 3.0 mm Portal venous phase: 70-s post-contrast delay, section thickness 3.0 mm	YES
Park [33]	4-, 8-, 16-, and 64- slice	Not available	120 mL Ultravist 370 (Iopromide)	***Bolus tracking*** **:** 100 HU at abdominal aorta 64-slice/16-slice/8-slice Early arterial phase: 6-s post-threshold (23-s post-contrast delay), slice thickness 3 mm. Late arterial phase: 5–9-s interscan delay between early and late phases, (37-45-s post-contrast delay); slice thickness of 3 mm. Venous phase: 70-s post-threshold: slice thickness of 3 mm 4-slice Early arterial phase: 6-s post-threshold (23-s post-contrast delay), slice thickness of 3.2 mm. Late arterial phase: 5–9-s interscan delay between early and late phases, (37-45-s post-contrast delay); slice thickness of 3.2 mm. Venous phase: 70-s post-threshold: slice thickness of 3.2 mm.	YES
Satoi [34]	4-slice	Not available	Not available	Arterial and portal phase; slice thickness 1.25 mm	NO
Croome [35]	Not available	Not available	Not available	Not available	NO
Grieser [36]	4-, 8-, 16-, and 64-slice	Not available	100 mL Ultravist 370 (Iopromide)	Bolus threshold: aortic enhancement above 100 HU Early arterial phase: 4-s post-threshold delay (20-s post-contrast delay) Early portal venous phase: 20 s delay from the beginning of the arterial phase (40-s post-contrast delay) Venous phase: 60-s delay from the beginning of the arterial phase (80-s post-contrast delay)	YES
Grossjohann [37]	64-slice	Not available	Not available	Not available	NO
Kaneko [38]	4-, 16-, and 64-slice	Not available	120–150 mL Omnipaque 350	4-slice Pancreas phase delay: 45 s, Slice thickness 1.25 mm, Portal venous phase: 60-70s, Slice thickness 1.25 mm, 16 and 64 slice Pancreatic phase: **bolus tracking** with trigger at 150 HU; slice thickness: 0.75 mm Portal venous phase: 10-s delay after pancreatic phase, slice thickness: 0.75 mm	YES
Lee [39]	4-slice	No oral contrast agent	150 mL Ultravist 370 (Iopromide)	***Bolus triggering*** **:** 100-HU enhancement of the descending aorta Arterial phase: 5-s post-threshold delay; reconstruction interval of 5 mm Portal venous phase: 72-s post-contrast delay; reconstruction interval of 5 mm	YES
Koelblinger [40]	64–slice	20 min before CT exam: 1000 mL water orally	150 mL Iomeron 300 (Iomeprol)	***Bolus triggering*** **:** threshold of 100 HU in the abdominal aorta. Pancreatic parenchymal: 25 –s post-threshold, slice thickness 3 mm Portal venous phase: 23-s after pancreatic phase, section thickness 3 mm	YES
Fang [41]	64 slice	Not available	80-100 mL Iopamiron	***Bolus tracking***: 100 HU in the diaphragmatic section of the abdominal aorta Arterial-phase: 8-s post-threshold slice thickness 0.67 mm Portal venous phase: 60-s post-threshold; slice thickness 0.67 mm	YES
Khattab [42]	64-slice	1000 mL of mixed water and contrast orally	100 mL Omnipaque 350	***Bolus triggering*** **:** 110 HU in the aorta at corresponding level of superior mesenteric artery. Pancreatic arterial phase: 20-s post-threshold delay; section width of 1.5 mm Delayed venous phase: 50-s post-threshold delay; section width of 1.5 mm	YES
Yao [43]	Not available	Not available	Not available	Not available	NO
Cieslak [44]	Range: 16–64-slice	Oral contrast	100-150 mL	Portal OR Arterial and portal; 5.0-mm slice thickness	NO
Hassanen [45]	16-slice	600–800 mL water or water soluble contrast agent orally	100 mL Ultravist 370 (Iopromide)	***Bolus triggering*** **:** 110 HU in the abdominal aorta at the level of the celiac axis Late arterial phase: 10- s post-threshold delay; 3.0 mm Portal venous phase: 35-s post-threshold delay; 3.0 mm	YES
Iscanli [46]	16- or 64- slice	1000 mL water orally	90-110 mL Visipaque 320 (Iodixanol) or Ultravist 370 (Iopromide)	***Bolus tracking***: 180-HU enhancement on the proximal abdominal aorta Arterial phase: start automatically when maximum contrast reached Pancreatic phase: 45-s post-contrast delay: slice thickness: 1-1.25 mm, Portal phase: 65-s post-contrast delay: slice thickness: 1-1.25 mm	YES

^*^The execution of the CT was described in sufficient detail if type of scanner, the type, amount, and concentration of iv contrast and the different phases with scan delay were described

### Interpretation of CT

Methods of reconstruction and the experience of observers were poorly described. However, the criteria used for unresectability was described in most of the papers, except in three studies [30, 35, 45]. In three studies [24, 25, 41], only vascular invasion was used as criteria for unresectability; in other studies all criteria were used. The interpretation of CT was not described in sufficient detail due to missing information on description of reconstruction methods, number and experience of observers and the criteria for resectability/unresectablity. Whether CT interpretation was blinded was not clear in most of the studies. All data in details are given in Table 4.

**Table 4:** Interpretation of CT in included studies

Study author	Method of reconstruction*	Observers (number and experience)	CT criteria for resectability or unresectability	Interpretation described in detail†	Blinded interpretation of CT‡	Criteria defined§
Ellsmere [18]	Not available	4 abdominal radiologists independently	Criteria for resectability: 1. No distant metastasis; 2. A patent portal vein; 3. <50% arterial involvement	NO	YES	YES
Imbriaco [19]	VR	2 experienced abdominal radiologists independently	Criteria for unresectability: 1. Peripancreatic vascular invasion (defined as encasement of the celiac, hepatic, or superior mesenteric arteries or the portal or superior mesenteric vein) ¶; 2. Extrapancreatic invasion of adjacent tissues and organs other than duodenum; 3. Presence of hematogenous or distant lymph node metastases (other than peripancreatic nodes); 4. Signs of peritoneal carcinomatosis; 5. Distant lymph node exceeding 1.5 cm ¶ A vessel was considered to be involved if it showed a focal reduction in caliber, circumferential (>180°) encasement by tumour, or frank thrombosis. Involvement of the splenic artery or veins was not considered an absolute contraindication to resection unless the tumour extended into the splenoportal confluence or involved other splanchnic vessels.	NO	YES	YES
Karmazanovsky [20]	MPR and VR	Not available	Criteria for unresectability: 1. Invasion of any of the vascular structures (portal vein, superior mesenteric vein, and artery) 2. Liver metastasis	NO	UNCLEAR	YES
Li [21]	MPR and MIP and VR	2 observers in consensus (10 and 5 years of experience in pancreatic imaging)	Criteria for unresectability: 1. Vascular invasion; 2. Hepatic metastasis 3. Other metastatic signs.	YES	YES	YES
Phoa [22]	Not available	2 experienced abdominal radiologists independently, followed by consensus	Criteria for unresectability: 1. Tumour infiltration was present; 2. If any vessel (portal or superior mesenteric vein, hepatic artery, and the superior mesenteric artery) showed involvement of >180 degrees; 3. If tumour convexity was scored as Loyer grade D or E ¶ 4. Liver metastases; 5. Distant lymph nodes larger than 1 cm ¶ Vascular invasion according to Loyer grade ^1^ Grade A: fat plane visible between tumour and vessel; Grade B: normal pancreatic tissue between tumour and vessel; Grade C: tumour adjacent to vessel with a contour convex towards the vessel; Grade D: tumour adjacent to vessel with a contour concave towards the vessel, Grade E: circumferential involvement of vessel; Grade F: thrombosis or occlusion of vessel.	NO	YES	YES
Imbriaco [23]	Not available	2 experienced radiologists independently	Criteria for unresectability: 1. Peripancreatic vascular invasion (defined as infiltration of the coeliac trunk, hepatic artery, superior mesenteric artery or portal vein, and superior mesenteric vein) ¶; 2. Extrapancreatic invasion of adjacent tissues 3. Hematogenous or lymph node metastasis (>1.5 cm) or peritoneal carcinomatosis. ¶ The vessel was considered involved when its circumference was reduced more than >180° by the tumour or by evident thrombosis. Involvement of the splenic artery or vein was not considered an absolute contraindication to surgery unless the tumour extended to splenic-mesenteric-portal confluence.	NO	YES	YES
Tamm [24]	Not available	3 abdominal radiologists independently	Criteria for unresectability: 1. Primary tumour involving the superior mesenteric or celiac arteries, involvement of the common hepatic artery, M1 disease, or occlusion of the superior mesenteric vein and/or portal vein.	NO	YES	YES
Kala [25]	Not available	Not available	Criteria for unresectablity: 1. Tumour invasion to coeliac trunk, to common hepatic artery, superior mesenteric artery, superior mesenteric vein, portal vein, inferior caval vein, and aorta.	NO	UNCLEAR	Yes
Olivie [26]	MPR and curved MPR	Not available	Criteria for unresectability: 1. Liver metastases; 2. Peritoneal carcinomatosis; 3. Tumour infiltration in contact with more than 180° of the circumference of the walls of major arteries (celiac trunk, hepatic artery, superior mesenteric artery) and involvement of more than 180° of the circumference of the portal vein or the superior mesenteric vein.	NO	YES	YES
Smith [27]	MPR and MIP and VR	2 radiologist independently followed by consensus	Criteria for unresectability: 1. Metastases 2. Vascular encasement: Lu grade 3 or 4¶ 3. Vascular occlusion 4. Direct invasion of bowel, spleen, and mesentery (duodenum excluded) 5. Lymphadenopathy (nodes > 1 cm in short axis) outside the peripancreatic chain. ¶ Vascular involvement (for the superior mesenteric vessels, coeliac artery, and portal vein) was estimated using the Lu grade ^2^. Grade 0: no contiguity of tumour to vessel; Grade 1: tumour contiguous less than one quarter of circumference; Grade 2: between one quarter and one half; Grade 3: between one half and three quarters; Grade 4: greater than three quarters.	NO	UNCLEAR	YES
Zamboni [28] (initial clinical evaluation)	MPR and MIP and VR	2 readers: one radiology resident/abdominal fellow (pool of 30 readers, with 2–6 years of experience) and one abdominal radiologist (pool of eight readers, with 8–20 years of experience).	Criteria for unresectability: 1. Distant disease (hepatic metastases, distant lymph node metastases, peritoneal metastases), 2. Invasion of adjacent organs other than the duodenum 3. Major vascular invasion: Grade 2, 3, or 4¶ 4. Lymph nodes were considered metastatic if the short-axis diameter was 1 cm or larger. ¶ Vascular invasion Grade 0: normal, with a fat plane or normal pancreas between tumour and vessel; Grade 1: loss of fat plane between tumour and vessel, with or without smooth displacement of the vessel); Grade 2: flattening and/or slight irregularity of one side of the vessel; Grade 3: encased vessel with tumour extending around at least two sides (i.e., two thirds of the perimeter), altering its contour and producing concentric or eccentric lumen narrowing; Grade 4: at least one major occluded vessel.	YES	UNCLEAR	YES
Zamboni [28] (retrospective evaluation)		2 gastrointestinal radiologists in consensus (>20 and 4 years of experience)	Same as for initial interpretation	YES	YES	YES
Furukawa [29]	MPR	More than 1 diagnostic radiologist in conference	Criteria for unresectablity: 1. Extrapancreatic tumour spread, particularly into the celiac axis and the root of the superior mesenteric artery; 2. Para-aortic massive lymph node involvement [the short axis was greater than 1 cm in diameter or there were clusters of 3 or more smaller nodes (each < 1 cm)] 3. Presence of distant metastases and ascites; 4. Portal vein invasion (>90% direct contact).	NO	YES	YES
Klauss [30]	Curved MPR	2 radiologists in consensus (with 11 and 23 years of experience)	Not defined for resectability (only for vascular invasion)	NO	YES	NO
Shah [31]	Not available	Interpretated by an experienced multidisciplinary team	Criteria for unresectablity: 1. Extra-pancreatic disease such as liver, omental, or peritoneal metastases; 2. Bulky (>2 cm) celiac adenopathy; 3. Malignant ascites; 4. Loss of a patent portosplenic confluence, or 360° encasement of the portal or superior mesenteric veins, or 5. Any contact between the tumour and the hepatic artery or superior mesenteric artery.	NO	UNCLEAR	YES
Manak [32]	MPR	2 radiologists in consensus	Criteria for unresectability 1. Vascular involvement ¶ 2. Liver and peritoneal metastases 3. Distant lymph nodes metastases. Enlarged lymph nodes (>10 mm) were considered as metastatic. Distant lymph nodes metastases beyond the peripancreatic chains indicated unresectability ¶ Arterial invasion (celiac trunk, superior mesenteric artery and common hepatic artery) was defined as any direct contiguity tumour to artery with complete obliteration of a fat plane even if the contiguity was less than 50%. ¶ Venous invasion (superior mesenteric vein, splenic vein, and portal vein) was defined when the tumour showed contiguity to more than 50% of vein circumference.	NO	YES	YES
Park [33]	Not defined	2 abdominal radiologists independently (8 and 7 years CT and MRI experience)	Criteria for unresectability: 1. Distant metastasis: liver, peritoneum, para-aortic lymph nodes, or lung; 2. Invasion into peripancreatic arteries: celiac trunk, hepatic artery, or SMA; 3. Massive venous invasion of portal vein and/or SMV, i.e., tumour infiltration with thrombosis and obliteration of the vessel lumen involving a segment longer than 2 cm; and 4. Infiltration in adjacent organs: stomach, spleen, or colon.	YES	NO	YES
Satoi [34]	MPR	1 experienced hepatopancreatolobiliary surgeon and 1 consultant radiologist in consensus	Criteria for potential resectable: 1. Patients with no distant metastases or no tumour extension to a major peripancreatic artery defined as tumour ingrowth with > 50% vessel contiguity in the celiac trunk, common or proper hepatic artery, or superior mesenteric artery 2. Patients with tumours invading the portal vein but in the absence of extended obstruction of the portal vein to distal branches of the superior mesenteric vein 3. Patients with cancer in pancreatic body and tail with celiac trunk invasion and without SMA invasion	NO	UNCLEAR	YES
Croome [35]	Not available	Not available	Not available	NO	UNCLEAR	NO
Grieser [36]	MPR and curved MPR and MIP and VR and additional 3D	2 experienced radiologists independently (4 and 6 years of experience in abdominal MDCT)	Criteria for unresectability: 1. Arterial infiltration (celiac trunk, superior mesenteric artery, common hepatic artery), 2. Distant metastases, and 3. Peritoneal carcinomatosis 4. Additional criterion comprised non-manageable venous infiltration or thrombosis (portal vein, superior mesenteric vein).	YES	YES	YES
Grossjohann [37]	Not available	Not available	Criteria for resectability: 1. No invasion into larger surrounding vessels or surrounding organs; 2. Not included in a resected Whipple’s specimen; 3. No detection of liver metastases 4. No distant lymph node metastases 5. No peritoneal seeding.	NO	UNCLEAR	YES
Kaneko [38]	MPR	Experienced radiologists specializing in gastrointestinal radiology	Criteria for unresectability: 1. distant metastases to liver, peritoneum, omentum, or adjacent organs; 2. Invasion of a major peripancreatic vessel (celiac artery, hepatic artery, portal vein, and superior mesenteric artery and vein (Lu grade of 3 or greater) ¶ 3. Venous invasion causing thrombosis of the vein. ¶ Vascular involvement according to Lu ^2^: Grade 0: tumour not contiguous with vessel; Grade 1: tumour contiguous with less than a quarter of vessel circumference; Grade 2: between a quarter and one half; Grade 3: between one half and three quarters; Grade 4: greater than three quarters or with any evidence of focal vessel narrowing regardless of degree of contiguity.	NO	UNCLEAR	YES
Lee [39]	MPR and MIP and VR	2 radiologists independently (completed fellowship in gastrointestinal radiology)	Criteria for unresectability: 1. Extrapancreatic invasion of the adjacent tissues or organs other than the duodenum and spleen, 2. Peritoneal metastasis, 3. Distant metastases, 4. Vascular invasion ¶. ¶ We used the criteria for arterial invasion constituted encasement, vessel margin irregularity, or tumour incursion into the periarterial fat plane with the tumour lying in juxtaposition to the vessel. ¶ Venous invasion was considered present when the tumour caused venous occlusion, flattening or narrowing, apposition with concavity toward the vessel lumen, or a circumferential apposition greater than 180°.	YES	YES	YES
Koelblinger [40]	MPR and curved MPR	2 gastrointestinal radiologists independently (>10 years of experience in abdominal CT and MR imaging)	Criteria for unresectablity: 1. Vascular invasion: the main portal vein, the portal venous confluence, the superior mesenteric artery and vein, the celiac trunk, and the hepatic artery, Grade 3 or more ¶; 2. The presence of liver metastases, 3. Peritoneal carcinomatosis, 4. Adjacent organ infiltration, and 5. Pathologic lymph nodes ¶ Vascular invasion according to Li ^3^ Grade 0: no tumour contiguity; Grade 1: tumour contiguous with less than 90° vessel circumference; Grade 2: tumour contiguous between 90° and 180° circumference; Grade 3: tumour contiguous between 180° and 270° circumference; Grade 4: tumour contiguous with more than 270° circumference.	YES	YES	YES
Fang [41]	VR	2 radiologists for CTA 2 radiologists for 3D reconstructions	CTA criteria for unresectablity: 1. A tumour intricately associated with the celiac trunk and its main branches, the abdominal aorta, the inferior vena cava, the portal vein, the superior mesenteric artery, the inferior mesenteric vein such that there is no apparent space in between them; 2. A low-density tumour completely surrounding its neighbouring blood vessels without causing lumen changes; 3. A low-density tumour that causes occlusion or stenosis by vascular invasion. 3D Criteria for unresectability: The following vessels were evaluated: portal vein, superior mesentery artery, inferior vena cava, superior mesenteric vein, left renal vein, right renal vein, hepatic artery, celiac trunk, and abdominal aorta. Type IV and V were classified as unresectable Type IV: the primary tumour was attached to and compressed the large vessels and the vessels had a “moth-eaten” instead of smooth appearance. Type V: the large blood vessels were surrounded by the primary tumour, distortion of the large vessels was evident and significant dilatation of the small pancreatic vein could be observed.	NO	YES	YES
Khattab [42]	Curved MPR	2 radiologists in consensus	Criteria of unresectability: 1. Tumours which were larger than 2 cm in size (tumours less than 2 cm may be associated with favorable outcome 2. Presence of local metastasis (such as enlarged lymph nodes outside peripancreatic draining chains) or direct invasion of the surrounding organs with exclusion of the duodenum, or more, 3. Distant metastases (liver or pulmonary metastasis), 4. Infiltration of the walls of major vessels including (celiac trunk, hepatic artery, splenic artery, superior mesenteric artery and portal vein or the superior mesenteric vein) ¶ ¶ Arterial vascular involvement was estimated using the criteria by Lu et al ^2^. Grade 0–2 was considered operable, whereas grades 3 and 4 were considered radiologically inoperable. Grade 0: no contiguity of tumour to vessel; Grade 1: tumour contiguous less than one quarter of circumference; Grade 2: between one quarter and one half; Grade 3: between one half and three quarters; Grade 4: greater than three quarters. ¶ Venous invasion was defined as tumour-to-vessel circumferential contiguity of 50% or more. Tumour-to-vein circumferential contiguity of less than 50% was not considered venous invasion	NO	YES	YES
Yao [43]	Not available	Not available	Criteria for resectability: 1. No distant metastases; 2. Absence of tumour-induced occlusion of any aspect of the superior mesenteric-portal vein confluence; 3. Absence of tumour extension to the celiac axis, common hepatic artery and superior mesenteric artery.	NO	UNCLEAR	YES
Cieslak [44]	Not available	2 (1 surgeon and 1 expert radiologist) in consensus	Criteria for unresectability: 1. Vascular invasion: when exceeding 270 ^o^ of the circumference of the portal vein/superior mesenteric vein or exceeding 90 ^o^ of the circumference of the superior mesentery artery, celiac trunk, or hepatic artery; 2. Distant metastases	NO	YES	YES
Hassanen [45]	Not available	2 radiologist	Not available	NO	UNCLEAR	NO
Iscanli [46]	MPR and curved MPR MIP and VR	By one of two radiologists (5 and 12 years of experience in reading pancreatic imaging)	Criteria for unresectability: 1. Distant metastases to the liver, peritoneum, or omentum; 2. Direct invasion of the adjacent organs (except the duodenum); 3. Vascular invasion of a major peripancreatic vessel, according to Lu. A Lu grade 0 to 2 was considered operable, whereas grade 3 and above were radiologically inoperable. A degree of contact of the vessel with the tumour of 180° was considered indeterminate¶. ¶ Lu criteria ^2^ evaluate vascular involvement by the degree of contact of the vessel with the tumour: Grade 0: no contiguity of tumour to vessels; Grade 1: tumour contiguous less than one quarter (<90°) of vessel circumference; Grade 2: tumour contiguous between one quarter and one half (90°-180°); Grade 3: between one half and three quarters (180°-270°); Grade 4: greater than three quarters (>270°) or any evidence of focal vessel narrowing or irregularity on the vessel wall, regardless of degree of contiguity.	YES	UNCLEAR	YES

^*^MPR: multiplanar reformation; MIP: maximum intensity projection; VR: volume rendering

^†^The interpretation of the CT was described in sufficient detail on number and experience of observers and the criteria for resectability/unresectablity were given

^‡^whether the CT results were interpreted without knowledge of the results of the reference standard

^§^If resectability/unresectability criteria were pre-specified (yes, no, unclear)

^1^Loyer EM, David CL, Dubrow RA, Evans DB, Charnsangavej C. Vascular involvement in pancreatic adenocarcinoma: reassessment by thin-section CT. Abdom.Imaging 1996; 21:202-20

^2^Lu DS, Reber HA, Krasny RM, Kadell BM, Sayre J. Local staging of pancreatic cancer: criteria for unresectability of major vessels as revealed by pancreatic-phase, thin-section helical CT. AJR Am. J Roentgenol. 1997; 168:1439-1443

^3^Li H, Zeng MS, Zhou KR, Jin DY, Lou WH. Pancreatic adenocarcinoma: the different CT criteria for peripancreatic major arterial and venous invasion. J Comput. Assist. Tomogr. 2005; 29:170-175

### Reference standard and time interval between CT and reference standard

The interval time between CT and reference standard were only mentioned in ten studies [21, 26–28, 32, 36, 38, 42, 45, 46]. Most reference concerned surgery followed by resection. All details on reference standard and time interval between CT and reference standard are given in Supplement 2.

### Data on resectability

As most studies were retrospectively performed, data on 2 x 2 tables or 1 x 2 tables were available, for details see Table 5.

**Table 5:** Data on resectability by CT

Study author	Resectable according to CT		Unresectable according to CT		PPV	FP distributions
	Resectable according to reference standard (TP)	Unresectable according to reference standard (FP)	Resectable according to reference standard (FN)	Unresectable according to reference standard (TN)	TP/(TP + FP)	
Ellsmere [18]	22	14	1	7	61.1% (22/36)	Not available
Imbriaco [19] (Obs1)	8	2	1	29	80.0% (8/10)	Not available
Imbriaco [19] (Obs 2)	7	3	2	28	70.0% (7/10)	Not available
Karmazanovsky [20] (Potential resectable as resectable)	52	18	4	15	74.3% (52/70)	Liver metastases (18)
Li [21]	16	6	1	31	72.7% (16/22)	Invaded vessels (4)/Liver metastases (1) Peritoneal metastases (1)
Phoa [22]	26	10	15	20	72.2% (26/36)	Not available
Imbriaco [23] (Obs 1)	6	2	1	37	75.0% (6/8)	Not available
Imbriaco [23] (Obs 2)	6	3	1	36	66.7% (6/9)	Not available
Tamm [24] (Obs 1)	10	2	1	42	83.3% (10/12)	Liver metastases (1)/Peritoneal metastases (1)
Tamm [24] (Obs 2)	10	3	1	41	76.9% (10/13)	Liver metastases (1)/Peritoneal metastases (1)/Vascular invasion (1)
Tamm [24] (Obs 3)	10	6	1	38	62.5% (10/16)	Liver metastases (3)/Peritoneal metastases (1)/Vascular invasion (1)/Lung metastases (1)
Kala [25]	14	14	2	19	50.0% (14/28)	Not available
Olivie [26]	23	0	0	5 (palliative procedure)	100.0% (23/23)	Not applicable
Smith [27] (Equivocal as resectable)	10	14	1	8	41.7% (10/24)	Vascular invasion (9)/Infiltration beyond pancreas (3)/Liver metastases (1)/Other metastases (1)
Smith [27] (Equivocal as unresectable)	9	7	2	15	56.3% (9/16)	Vascular invasion (3)/Infiltration beyond pancreas (3)/Other metastases (1)
Zamboni [28] (Clinical evaluation)	78	10	0	26	88.6% (78/88)	Vascular invasion (1)/Liver metastases (5) Lymph nodes (2)/Peritoneal metastases (2)
Zamboni [28] (Retrospective evaluation)	78	2	0	34	97.5% (78/80)	Not available
Furukawa [29] (Probably unresectable as unresectable)	68	11	0	134	86.1% (68/79)	Peritoneal metastases (7)/Liver (3)/LN (1)
Klauss [30]	21	0	1	6	100% (21/21)	NA
Shah [31]	34	13	NA	NA	72.3% (34/47)	Metastases (9)/R2 resection (1)/Locally advanced (3)
Manak [32]	44	4	NA	NA	91.7% (44/48)	Vascular invasion (1)/Liver metastases (2)/Peritoneal metastases and lymph nodes (1)
Park [33] (Obs 1)	35	3	7	9	92.1% (35/38)	Not available
Park [33] (Obs 2)	35	4	7	8	89.7% (35/39)	Not available
Satoi [34]	29	3	NA	NA	90.6% (29/32)	Distant metastases (3)
Croome [35]	24	16	NA	NA	60.0% (24/40)	Liver metastases (3)/Peritoneal metastases (1)/Omental seeding (1)/Vascular invasion (10)/Vascular invasion and lymph nodes (1)
Grieser) [36] (Without 3D) (Obs 1)	32	6	1	31	84.2% (32/38)	Not available
Grieser [36] (With 3D) (Obs 1)	32	4	1	33	88.9% (32/36)	Not available
Grieser [36] (Without 3D) (Obs 2)	32	5	1	32	86.5% (32/37)	Not available
Grieser [36] (With 3D) (Obs 2)	32	3	1	34	91.4% (32/35)	Not available
Grossjohann [37]	10	9	2	8	53% (10/19)	Liver metastases (3)/Lymph node metastases (1)/Vascular invasion (5)
Kaneko [38] (Equivocal as resectable)	67	20	0	22	77.1% (67/87)	Liver metastases (4)/Vascular invasion (10)/Peritoneal metastases (6)
Kaneko [38] (Equivocal as unresectable)	67	12	0	30	84.8% (67/79)	Liver metastases (4)/Vascular invasion (4)/Peritoneal metastases (4)
Lee [39] (Obs 1)	35	6	4	11	85.4% (35/41)	Peritoneal metastases (2)/Liver metastases (1)/Vascular invasion (1)/Adjacent organs (2)
Lee [39] (Obs 2)	35	7	4	10	83.3% (35/42)	Not available
Koelblinger [40] (Obs 1)	13	2	2	6	86.7% 13/15	Carcinomatosis (1)/Lymph node (1)
Koelblinger [40] (Obs 2)	13	3	2	5	81.3% 13/16	Carcinomatosis (2)/Lymph node (1)
Fang [41] CTA	30	2	8	17	93.8% (30/32)	Vascular invasion (2)
Fang [41] 3D	38	0	0	19	100% (38/38)	Not Applicable
Khattab [42]	15	3	NA	NA	83.3% (15/18)	Vascular invasion (2)/Liver and lymph nodes (1)
Yao [43]	25	11	NA	NA	69.4% (25/36)	Bone metastases (2)/Lymph nodes (1)/Liver metastases (8)
Cieslak [44]	75	8	1	2	90.4% (75/83)	Distant metastasis (4)/Locally advanced (3)/Liver metastases (1)
Hassanen [45]	13	6	2	26	68.4% (13/19)	Vascular invasion (4)/Liver metastases (2)
Iscanli [46] (Equivocal as resectable)	62	21	0	41	75.7% (62/83)	Liver metastases (9)/Peritoneal metastases (3)/Vascular invasion (9)
Iscanli [46] (Equivocal as unresectable)	62	17	0	44	78.5% (62/79)	Liver metastases (9)/Peritoneal metastases (3)/Vascular invasion (5)

NA: Not Applicable; Obs 1: Observer 1; Obs 2: Observers 2; Obs 3: Observer 3

#### Publication bias

**Fig. 2:**
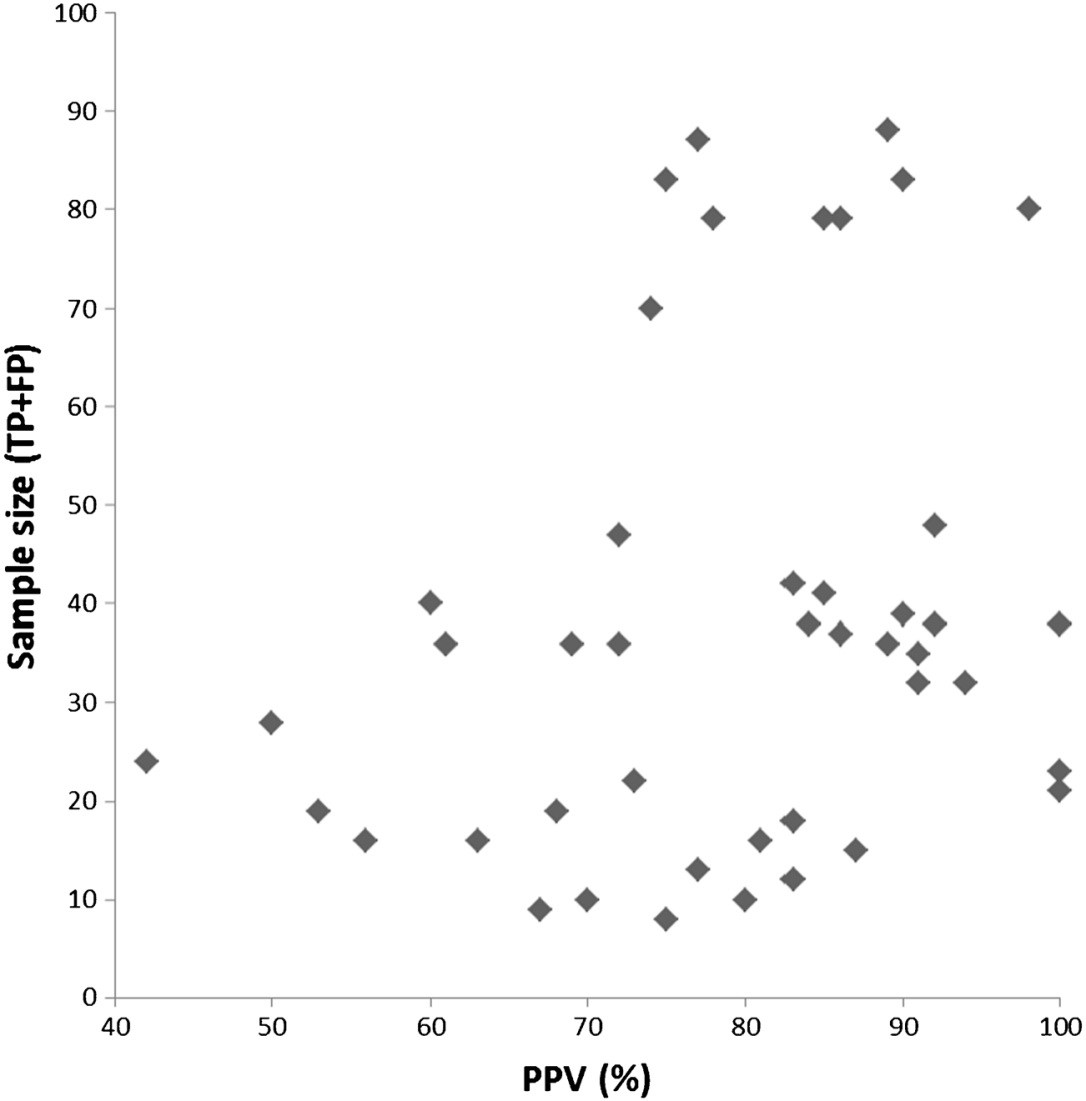
Funnel plot showing no relationship between sample size and PPV

The regression coefficient showed no significant relationship between sample size and PPV. The coefficient was 0.47 (95%CI: -0.06-0.99) with a *p*-value of 0.08 (see also Fig. 2).

#### Summary Positive Predictive Value (sPPV)

The I^2^ value was 68% (95%CI: 57-76%), indicating that data were heterogeneous. The sPPV was: 81% (95%CI: 75-86%). The false positives were mostly liver metastases, peritoneal metastases, or lymph node metastases. This means that these metastases were missed at CT (Table 7).

#### Exploratory analysis

Only description of CT technical features, description of interpretation of CT and blinded interpretation of CT had effect on summary PPV. All other items did not have an effect (all *p* > 0.05).

Studies where CT was described in details compared to studies where CT was not described in details: 85% (95%CI: 79-89%) and 72% (95%CI: 61-81%) respectively (*p* = 0.013).

Studied where CT interpretation was described in details compared to studies where CT interpretation was not described in details: 89% (95%CI: 83-93%) and 74% (95%CI: 67-80%), respectively (*p* = 0.004).

Studies where CT interpretation was blinded compared to studies where CT interpretation was not clear: 87% (95%CI: 81-91%) and 72% (95%CI: 63-80%), respectively (*p* = 0.001).

#### Subgroup analyses

Studies using bolus-triggering for CT scanning had a slightly higher sPPV compared to studies using fixed timing, respectively 87% (95%CI: 81-91%) and 78% (95%CI 66-86%); however, with a *p*-value of 0.077.

No significant difference was observed between studies including both pancreatic and portal phases vs. studies including only portal phase, respectively 84% (95%CI:78-88%) and 75% (95%CI: 60-85%), *p* = 0.153.

No difference was seen between studies taking all criteria into account compared to studies taking only vascular invasion as criteria for unresectablity, respectively 81% (95%CI: 76-86%) and 81% (95%CI: 45-96%), *p* = 0.984.

## Discussion

This meta-analysis showed a sPPV of 81% (95%CI: 75-86%) for predicting resectability by CT. This means that the percentage of patients falsely undergoing surgical exploration is 19%. The false positives (resectable on CT while unresectable on reference standard) for resectability were mostly distant metastases such as liver metastases, peritoneal metastases, or lymph node metastases.

Surprisingly, when checking the subgroup analyses, no significant difference was found between studies including both pancreatic and portal phases (sPPV: 84%) vs. studies including only portal phase (sPPV: 75%). One would except that if both phases are combined, the number of false positive would be significantly reduced, as both phases has a complementary role in the staging of pancreatic cancer [3–6]

The pancreatic phase is the most important phase for detecting and staging a pancreatic tumour. In this phase there is optimal attenuation difference between the hypodense tumour and the normal enhancing pancreatic parenchyma. This phase is not only adequate in detecting primary tumour, but also in delineating periarterial tumour spread in relation to the celiac artery, celiac artery branches, and mesenteric arteries and veins as well (local staging).

The subsequent portal phase has a scan-delay of 70-80 s. At that moment the normal liver parenchyma will enhance optimally, because normal liver cells get 80% of their blood supply through the portal venous system. Liver metastases do not get their blood supply from the portal venous system and will be seen in this phase as hypodense lesions. This phase is, therefore, accurate for detection of liver lesions and is also used for the overall assessment of the abdomen to look for lymph nodes and peritoneal metastases.

We found no significant difference between both phases and only portal phase. This might be explained by the low number of studies using only portal phase, the heterogeneity within data or because the portal phase is also helpful for local staging of the tumour (primary goal of the pancreatic phase).

Based on the higher PPV, frequently used, and the complementary role of both phases, the use of both phases should be continued for evaluation of diagnosis, local staging (vascular invasion), and evaluation of metastatic disease.

Also, no differences were seen between studies taking all criteria (vascular invasion, liver metastases, peritoneal metastases, or lymph node metastases) into account compared to studies taking only vascular invasion as criteria for unresectablity. We expected less false positives when using all criteria, but the sPPV estimates were comparable 81% and 81%.

Most false positives for resectability in both subgroups were due to the presence of distant metastases such as liver metastases, peritoneal metastases, or lymph node metastases. This means that r distant metastases are missed by radiologists even if they are paying attention. In none of the studies, however, was the interpretation of liver or peritoneal metastases defined, and in general it is known that the role of imaging in detecting metastatic lymph nodes is still disappointing. This has also been shown in a recent published systematic review on the diagnostic accuracy of CT in assessing extra-regional lymph node metastases in pancreatic and peri-ampullary cancer, with a mean summary sensitivity of 25% [47].

Even though the distant metastases are missed even if paying attention, these features should be taken into account in the interpretation of CT for determining resectability and thereby defining criteria for especially the peritoneal and lymph node metastases, in order to further reduce the number of unnecessary surgical explorations.

Studies using bolus triggering had a slightly higher sPPV compared to studies using fixed timing, respectively, 87% and 78%; however, with a *p*-value of 0.077. In general study design and methodological criteria were poorly described. Most of them did not have effect on the outcome, except the description of CT technical features, description of CT interpretation and blinded interpretation of CT. For all these three items, it seems that in case the criteria were clearly described the sPPV raised. The role of these items also has been studied by different methodological group and was found to be relevant and, therefore, STARD 1 and STARD 2 have been developed on optimal reporting diagnostic accuracy studies [48, 49].

So far different reviews have been published on the diagnostic value of CT in evaluating vascular invasion [1, 2] and focusing on vascular invasion with promising results. Only one review published in 2005 evaluated the role of CT in determining resectability, however, they reported summary sensitivity and specificity [50] and not on the positive predictive value of CT. Several studies have been published on this topic after 2005.

We included all studies, hence also studies including a minor portion of patient with other cancer than adenocarcinoma. But most of the patients included were patients with adenocarcinoma. In addition, although the fixed scanning is an older method, there are still centres using this technique and, therefore, we also included these studies.

Most studies were retrospectively performed, and therefore, we were also able to include data on the patients with unresectable tumours on CT. But we did not summarize the negative predictive value (predictive value for unresectability), as it is known that these predictive values are high and in daily practice only patients with (potentially) resectable tumours on CT will undergo reference standard such as surgical exploration, and therefore, no data on reference standard by surgical exploration will be available in a prospective settings. Only one study was performed prospectively and all patients were verified by surgery/follow-up [19].

Summary positive predictive value (sPPV) of CT for determining resectability is high, however, still missing a significant number of patients with distant metastases such as liver metastases, peritoneal metastases, or lymph node metastases. In the paper of Allen et al [7], the false positive could be reduced from 40% to 17% using laparoscopy. Comparable findings are found in our meta-analysis when using only CT. However, a false positive rate of 19% is still high. In several studies the value of PET-CT has been studies in the staging of pancreatic cancer [8–11, 43, 51, 52] and showed to have an additional role in the staging, and thereby even reducing the number of unnecessary surgical exploration [11, 43, 51, 52].

Recommendation is to perform an additional imaging in the patients with (potentially) resectable pancreatic tumours to reduce the number of unnecessary surgical exploration.

## Electronic supplementary material

Below is the link to the electronic supplementary material.

ESM 1 (DOC 76 kb)
