# Supplementary material for: Contrast-enhanced CT in determining resectability in patients with pancreatic carcinoma: a meta-analysis of the positive predictive values of CT
Source: Eur Radiol. 2017 Jan 16;27(8):3408–35. doi: 10.1007/s00330-016-4708-5 (PMC5491588; doi:10.1007/s00330-016-4708-5)
Supplement: Supplementary file 1 — (DOC 76 kb) [file 330_2016_4708_MOESM1_ESM.doc]

**Supplement 1. Results of the search strategy**

| **MEDLINE** | **Search terms** | **Number of hits** |
| --- | --- | --- |
| #1 | Search "Pancreatic Neoplasms"[Mesh] | 56,799 |
| #2 | Search "Tomography, X-Ray Computed"[Mesh] OR "Multidetector Computed Tomography"[Mesh] OR "Four-Dimensional Computed Tomography"[Mesh] OR "Spiral Cone-Beam Computed Tomography"[Mesh] OR "Cone-Beam Computed Tomography"[Mesh] OR "Tomography Scanners, X-Ray Computed"[Mesh] OR "Tomography, Emission-Computed"[Mesh] OR "Tomography, Spiral Computed"[Mesh] OR "Tomography, Emission-Computed, Single-Photon"[Mesh] | 376,593 |
| # 3 | #1 AND #2 | 5421 |
| #4 | # 3 AND (Limit Human and **Publication date JAN2005- JUN 2015** | 2526 |

| **EMBASE** | **Search terms** | **Number of hits** |
| --- | --- | --- |
| #1 | pancreatic cancer.mp. or *pancreatic cancer/ | 41,252 |
| #2 | computed tomography.mp. or *computer assisted tomography/ | 278,942 |
| #3 | | #1 AND #2 | | --- | | 1492 |
| #4 | #3 AND Limit human AND publication date form 2005-2015 | 970 |

**Supplement 2. Reference standard and time interval between CT and reference standard.**

| **Study author** | **Interval between CT and reference standard** | **Proportion of study group undergoing reference standard** | **Appropriate interval between CT and reference standard*** | **Composition of reference standard** | **Reference standard correctly classify target condition**‡ |
| --- | --- | --- | --- | --- | --- |
| Ellsmere [18] | Not available | 44/44 | UNCLEAR | Surgery and resection | YES |
| Imbriaco [19] | Not available | 40/40 (malignant) | UNCLEAR | Surgery and resection and follow-up | YES |
| Karmazanovsky [20] | Not available | 89/89 | UNCLEAR | Surgery, palliative surgery and resection | YES |
| Li [21] | Within 2 weeks | 54/101 patient with adenocarcinoma | YES | Surgery and resection | YES |
| Phoa [22] | Not available | 71/71 | UNCLEAR | Surgery and resection | YES |
| Imbriaco [23] | Not available | 46/46 (malignant) | UNCLEAR | Surgery and resection | YES |
| Tamm [24] | Not available | 55/55 | UNCLEAR | Surgery and resection and follow-up and consensus (CT including) | NO |
| Kala [25] | Not available | 49/55 (49 patients with judgeable CT) | UNCLEAR | Surgery and resection | YES |
| Olivie [26] | Mean 22.4 days (range 3- 55 days) | 28/28 (study group) | NO | Surgery and resection | YES |
| Smith [27] | Within 3 weeks | 33/33 (study group) | YES | Surgery and resection | YES |
| Zamboni [28] | Range 1–73 days | 114/114 | NO | Surgery and resection | YES |
| Furukawa [29] | Not available | 213/213 | UNCLEAR | Surgery, resection, additional imaging with MRI, EUS, ERCP, and follow-up | YES |
| Klauss [30] | Not available | 28/35 (35 adenocarcinoma) | UNCLEAR | Surgery and resection | YES |
| Shah [31] | Not available | 47/88 | Unclear | Laparoscopy, surgery, and resection | YES |
| Manak [32] | 2 to 14 days (mean 5 ± 1.9 days) | 48/48 (study group) | YES | Surgery and resection | YES |
| Park [33] | Not available | 54/54 | Unclear | Surgery and resection | YES |
| Satoi [34] | Not available | 43/80 | Unclear | Surgery and resection | YES |
| Croome [35] | Not available | 55/96 (patients undergoing CT)) | UNCLEAR | Laparoscopy, surgery, and resection | YES |
| Grieser [36] | Mean 18 days (± 25) days. | 70/70 malignant (60 adenocarcinoma) | NO | Surgery and resection | YES |
| Grossjohann [37] | Not available | 29/44 (44 malignant adenocarcinoma) | UNCLEAR | Surgery and resection | YES |
| Kaneko [38] | 20 days (median) and 24.7 days (mean) | 109/109 (final group) | NO | Surgery and resection | YES |
| Lee [39] | Not available | 56/56 (all patients underwent surgery) | UNCLEAR | Surgery and resection | YES |
| Koelblinger [40] | Not available | 23/43 (43 pancreatic cancer) | UNCLEAR | Surgery and resection | YES |
| Fang [41] | Not available | 57/57 (57 pancreatic carcinoma) | UNCLEAR | Surgery and resection | YES |
| Khattab [42] | within 7–25 days (with mean 14.5 days) | 18/39 | YES | Surgery and resection | YES |
| Yao [43] | Not available | 36/36 (all patients underwent surgery, FU) | UNCLEAR | Surgery and resection/Follow-up and consensus | NO |
| Cieslak [44] | Not available | 86/86 (all patients underwent surgery) | UNCLEAR | Surgery and resection | YES |
| Hassanen [45] | Less than 2 weeks | 47/47 (study group) | YES | Surgery (including bypass) and resection | YES |
| Iscanli [46] | Between 3 and 21 days | 124/124 (124 patients underwent surgery) | YES | Surgery and resection | YES |

* Whether there was an appropriate interval (< 1 months for surgery/histology/aspiration etc., and < 12 months for FU) between CT and reference standard (yes, no, unclear);

‡ Whether the reference standard was likely to correctly classify the target condition (yes, no, unclear). In case of consensus including CT, the reference standard was assessed as not correctly classifying the target condition
